# Association of Urinary Lipocalin-2 with Lupus Nephritis

**Published:** 2013-09

**Authors:** Farzaneh Sharifipour, Abbasali Zeraati, Maryam Sahebari, Mohammadreza Hatef, Masih Naghibi, Zahra Rezaieyazdi, Mahmoud Mahmoudi, Amir Abbas Azarian, Zahra Mirfeizi, Katayoun Samadi

**Affiliations:** 1 **Imam Reza Hospital, Mashhad University of Medical Sciences, Mashhad, Iran**; 2 ** Rheumatic Diseases Research Center (RDRC), Mashhad University of Medical Sciences, Mashhad, Iran**; 3 ** Immunology Research Center (IRC), Mashhad University of Medical Sciences, Mashhad, Iran**; 4 ** Vice Chancellor for Research Office, Mashhad University of Medical Sciences, Mashhad, Iran**; 5 **Mashhad University of Medical Sciences, Mashhad, Iran**

**Keywords:** Lupus, Nephritis, SLE, Urinary Lipocalin- 2

## Abstract

***Objective(s):*** Lupus nephritis (LN) is the main cause of mortality and disability in systemic lupus erythematosus (SLE) patients. Therefore, utilizing a reliable and non-invasive method for serial measurements of renal function seems to be necessary. The aim of this study was to evaluate the role of urinary lipocalin-2 as a biomarker of renal involvement in SLE patients**.**

***Materials and Methods:*** Fifty two lupus patients in this cross sectional study were divided into two groups: patients with and without nephritis. For each group, urinary lipocalin-2, values were measured and reported according to urinary lipocalin-2/creatinine. Urinary lipocalin-2/creatinine sensitivity and specificity for identifying biopsy-proven nephritis were calculated, and a receiver operating characteristic (ROC) curve was constructed.

***Results*** *:* The mean urinary lipocalin-2/creatinine value of patients with biopsy-proven LN was 2.99 ± 4.1 ng/mg, and in non-LN patients was 1.16 ± 1.27 ng/mg. Urinary lipocalin-2/creatinine levels in LN patients were significantly higher than those in non-LN patients (*P*- Value = 0.03). In LN patients, urinary lipocalin-2/creatinine significantly correlated with proteinuria (r = 0.68; *P* = 0.0001). Using a cutoff value of 0.896 ng/mg, urinary lipocalin-2/creatinine had a sensitivity of 89.7% and a specificity of 39.1% for identifying SLE patients with biopsy-proven LN. The area under the ROC curve was 0.664 ± 0.076 with a 95% confidence interval of 0.52-0.81 (*P*=0.04). Analysis of variance showed that urinary lipocalin-2/creatinine is the same in different classes of LN (*P*-value=0.28).

***Conclusion:*** An important clinical conclusion is that measurement of urinary Lipocalin-2 may result in earlier diagnosis of LN.

## Introduction

Systemic lupus erythematosus (SLE) is characterized by the presence of autoantibodies against a variety of self antigens. Kidney involvement is a major concern in SLE and is blamed for a large degree of morbidity and mortality in patients. Unfortunately, the clinical management of nephritis being an autoimmune disease is still a great challenge because of its heterogeneous classification and unpredictable course (1). It is obvious that early diagnosis and treatment of lupus nephritis is associated with better disease outcome (2). Hence, it would be very beneficial if one could detect the presence of nephritis early in disease. Although serial renal biopsies may be ideal in close monitoring of progression of renal diseases, this may be practically difficult and is not without complications (3). Clearly, there is an urgent need for a "surrogate" marker of renal involvement to predict the onset of immune nephritis and to monitor its progression in lupus. Renal biopsy is usually suggested as the ‘gold standard’ for diagnosis and histological classification of LN (4). Nonetheless, renal biopsy is associated with important problems. Sampling bias appears a prominent problem because renal pathology may not be uniform in LN (5). The lipocalin protein family is a large group of mostly secreted soluble proteins that bind hydrophobic ligands and act as transporters, carrying small molecules to specific cells. Lipocalin-2, also known as neutrophil gelatinase-associated lipocalin (NGAL) is expressed in neutrophils and in low levels in the kidneys that are normally exposed to microorganisms and is upregulated during inflammation (6). There are many scenarios where lipocalin measurement could be useful, including haemolytic uraemic syndrome, acute tubular necrosis, acute renal failure following cardio-pulmonary bypass and chronic kidney disease (7). For the current study, we hypothesized that urinary lipocalin may also represent a novel biomarker for the identification and quantization of LN. Previous research suggests that lipocalin is a high-quality renal biomarker of acute kidney injury (8), while its usefulness in SLE is unclear. The aim of our study was to evaluate the role of urinary lipocalin-2II as a biomarker of renal involvement and nephritis in SLE. 

**Table 1:** Baseline characteristics of patients with and without lupus nephritis

Characteristics	With lupus nephritis	Without lupus nephritis	*P*-value
Number of participants	29	23	-
Female gender (%)	23(79.3)	21(91.3)	0.112
Age,y	26.60 ± 6.14	26.11 ± 5.48	0.782
Positive Anti dsDNA (%)	41.4	0	0.001*
Low C3 (%)	71.4	30	0.008*
Low C4 (%)	81	45	0.02*

## Materials and Methods

 In this cross sectional study which was conducted from July 2009 to September 2011, 52 SLE patients (29 LN patients and 23 patients without nephritis) were recruited consecutively. The study was approved by Ethics Committee of Mashhad University of Medical Sciences. All Patients gave written informed consent before entering the study. They were diagnosed according to the American College of Rheumatology (ACR) diagnostic criteria. As part of routine medical care during the study, patients with LN underwent renal biopsy. Renal biopsy was done when hematuria, increasing proteinuria or an increasing serum creatinine level, which is considered as active lupus nephritis was presented. All biopsies were classified according to the modified WHO classification into six classes, i.e. normal, mesangial, focal segmental, diffuse proliferative, membranous and advanced sclerosis. 

 Generally, patients with diabetes mellitus, malignancies and those with a diagnosis of overlap syndrome were excluded. In group without LN, patients with renal insufficiency from non-lupus-related causes were also excluded. Among the patients with LN, those undergoing hemodialysis were excluded. 

All patients underwent routine laboratory assessments. We obtained 5 ml blood samples from each patient. Blood samples were obtained for determination of serum C3, C4, anti nuclear antibody (ANA), anti-dsDNA.

***lipocalin-2/NGAL quantitative determination in urine samples***

 Urine spot Samples were stored in -70^O^C until use. Lipocalin-2/NGAL concentrations were determined using human lipocalin-2/NGAL ELISA kit (R&D Systems Inc., Minneapolis, MN) according to the manufacturer’s instruction. The intra-assay and inter-assay coefficients of variation were 3.7 and 5.9%, respectively. The level of lipocalin-2 was reported according to urinary lipocalin-2/creatinine expressed as ng/ml. A complete urine analysis and 24 hr urinary protein collection were obtained for each patient. 

***Statistical analysis***

Statistical analysis was performed with SPSS for windows version 11.5 (SPSS Inc., Chicago, IL, USA). Mean and standard deviations were used to express quantitative data. Comparison between means of the two groups was done by using Student's t-test. 

Correlation between urinary lipocalin-2/creatinine and 24 hr urine protein secretion were analyzed using Pearson`s correlation coefficient. *P-* values of <0.05 were considered as statistically significant. Urinary lipocalin-2/creatinine sensitivity and specificity for identifying biopsy-proven nephritis were calculated, and a receiver operating characteristic (ROC) curve was constructed.

## Results

**Figure 1:**
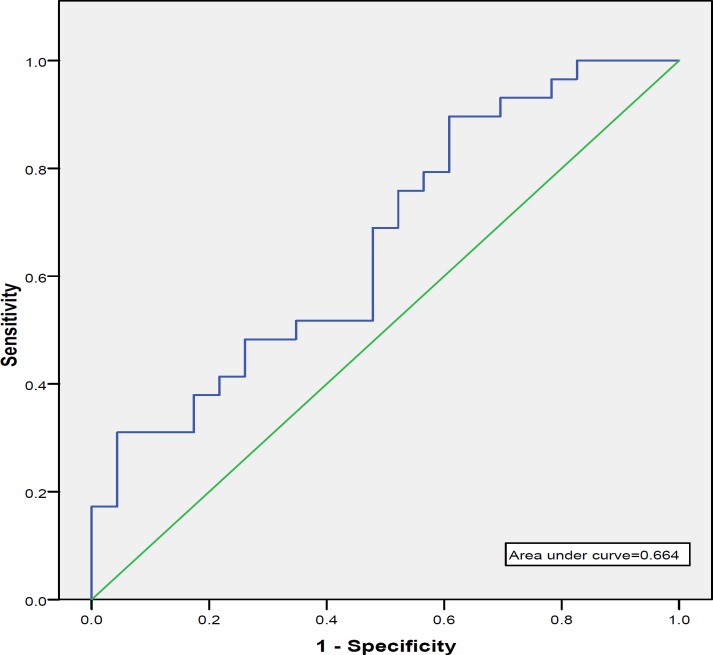
Receiver operating characteristic (ROC) curves of urinary lipocalin-2/creatinine for detection of lupus nephritis

**Figure 2:**
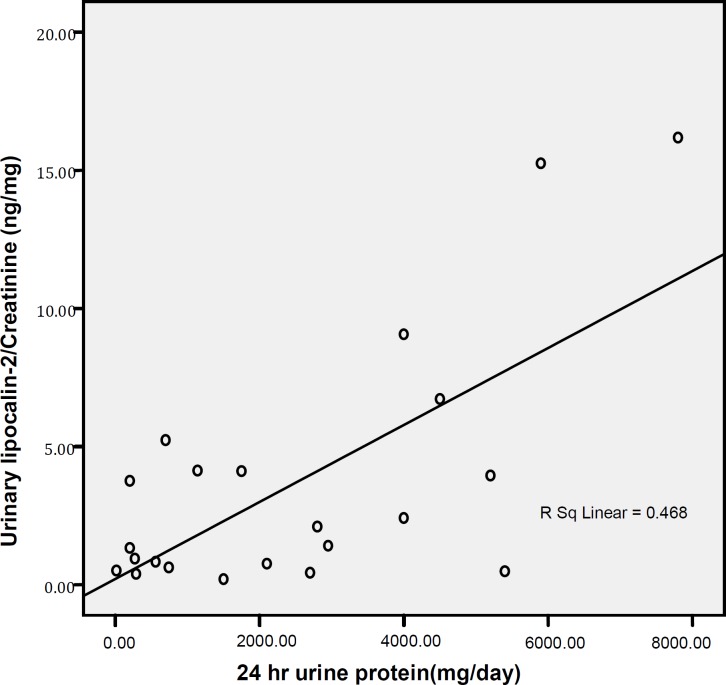
Scatter plot representing relationship between urinary lipocalin-2/creatinine and 24 hr urine protein in group with lupus nephritis

There were 29 patients (6 males and 23 females) in group with LN and 23 patients (2 males and 21 females) in group without LN. Forty four patients were females with a mean age of 26.71± 6.12 and 8 patients were males with a mean age of 24.71± 3.81. Demographics and clinical data of the patients with and without nephritis are shown in Table 1. ANA was positive in all patients. Anti dsDNA level in 41.5% of the SLE patients with nephritis was more than 800 IU, while, all of patients without nephritis had anti dsDNA results lower than 800 IU. There were no significant differences regarding age (*P*-value= 0.782), and sex(*P*- value= 0.38) between the two groups. C3 and C4 levels were significantly lower in patients with nephritis than in patients without nephritis. The mean urinary lipocalin-2/creatinine level of patients with biopsy-proven LN was 2.99 ± 4.1 ng/mg, while that of non-LN patients was 1.16 ± 0.425 ng/mg (Figure 1​). There was a significant difference in urinary lipocalin-2/creatinine levels between the two SLE groups (*P*-value = 0.03). In patients with nephritis, urinary lipocalin-2/creatinine significantly correlated with proteinuria (r = 0.684; *P* = 0.0001) (Figure 2​). Renal biopsy of the patients with nephritis showed mesangial disease (Class II, 9 cases), focal proliferative glomerulonephritis (Class III, 8 cases), and diffuse proliferative glomerulonephritis (6 cases); in patients with lupus nephritis, all groups of subjects had similar urinary lipocalin-2/creatinine ratios. To quantify the diagnostic utility of urinary lipocalin-2 as marker of nephritis in patients, we constructed an ROC curve (Figure 3), using urinary lipocalin-2/creatinine in patients with LN proven by biopsy (the current gold standard) and patients without nephritis. Using a cutoff value of 0.39 ng /mg, urinary lipocalin-2/creatinine had a sensitivity of 89.7% and a specificity of 39.1% for identifying patients with biopsy-proven nephritis. Positive predictive values (PPV) and negative predictive value (NPV) were estimated 65% and75% respectively. The area under the ROC curve was 0.664 ± 0.076 with a 95% confidence interval of 0.52 to 0.81 (*P*=0.04).

## Discussion

In the present study, we found that urinary lipocalin-2/creatinine level was higher in patients with lupus nephritis compared to those without nephritis and significantly correlated with proteinuria. Urinary lipocalin-2/creatinine had high sensitivity and low to moderate specifity for predicting LN in patients with SLE. One thing that is certain is that long-term survival in SLE can be improved with early diagnosis (and treatment) of LN. It is thus essential to identify new biomarkers to predict, diagnose and monitor LN. Since urinary biomarkers are more easily obtainable with much less risk to the patient than repeat renal biopsies, there has been tremendous need to identify urinary biomarkers with potential diagnostic value in lupus. 

Recently, lipocalin has been emerging as a novel biomarker of renal injury from several etiologies. Previous research suggests that lipocalin is a high-quality renal biomarker of acute kidney injury (9), while its usefulness in SLE is unclear.

 Our result is similar to that of Pitashny *et al* (10), who found significantly higher levels of urinary lipocalin-2 in SLE individuals in the presence of nephritis than in its absence. In a study by Brunner *et al*, urinary lipocalin-2 levels were found to be higher in pediatric patients with SLE compared to juvenile idiopathic arthritis (11). There are important differences between pediatric populations and adult populations. Adults tend to have many more comorbid health issues that could influence biomarker levels, so biomarkers such as NGAL typically have much better predictive and discriminatory value in children than in adults. Children as such tend to be a more pure experimental system.

 The result agrees with that reported by Bolignano *et al*, who also showed that urinary lipocalin concentrations correlated with renal function and a strong correlation existed between urinary lipocalin and the presence of proteinuria  (12). While the most likely source of urinary lipocalin in acute kidney injury is the distal tubules (13), the evidence of a strict correlation between urinary lipocalin and the degree of proteinuria may result from increased glomerular protein loss and disturbed reabsorption in the proximal nephron segment in addition to increased intrarenal production, so in response to large amounts of filtered protein due to a glomerular capillary leak, it is possible that urinary lipocalin is released from the renal tubular epithelium (14). 

 Our data supports the previous findings that lipocalin levels do not differ with patient age and sex (15). Among previous reports, Koura *et al*, 2011 (16) pointed out that children and adolescents with LN had significantly higher lipocalin levels than did healthy controls or those with juvenile idiopathic arthritis. Lipocalin levels were strongly correlated with renal disease activity but not with extra renal disease activity score. Although previous results firmly support the notion that lipocalin is very sensitive and specific for identifying childhood-onset SLE patients (14), we found that urinary lipocalin-2/creatinine had high sensitivity and low to moderate specificity for predicting LN in our adult patients with lupus. Some of the problems in the clinical use of lipocalin may include its nonspecific nature since urinary lipocalin levels also increase after various other types of kidney injury, including ischemic and toxic injuries. 

Although we did not find a difference in urinary lipocalin-2/creatinine ratio between different types of LN, this study was not powered to answer this question conclusively. Since different classes of LN, most notably WHO Class V LN, respond differently to treatment and may diverge in their pathogenesis, it will be interesting to see whether urinary lipocalin is more or less predictive of disease in different classes of nephritis in a larger cohort of biopsied patients. Interestingly, in paediatric lupus higher levels of urinary lipocalin were seen in patients with Class IV nephritis compared to patients with Class V nephritis (17). Patients with active renal disease recorded also significantly higher urinary lipocalin levels than lupus nephritis patients with inactive renal disease; and finally lipocalin was strongly correlated with the renal and not with the extrarenal disease activity score, supporting the notion that lipocalin could be a new renal biomarker in children and adolescents with biopsy proven lupus nephritis. Results of other routine tests used to evaluate renal function, such as serum creatinine and urine sediment, vary not only with LN activity but also with the presence of renal damage. Serological markers for systemic lupus activity, including anti-dsDNA antibodies, and complement components C3 and C4 were significantly different between two groups of patients with and without LN. In human SLE, the timing of an increase in urinary lipocalin-2 in relation to a rise in anti-dsDNA autoantibodies and decrease in C3 and C4 levels is still unknown and comparative studies between urinary lipocalin-2 and current disease activity markers such as ds-DNA and complement are also encouraged in future. 

 Furthermore, we will need to determine not only whether lipocalin-2 correlates with measures of disease activity and severity, but also whether its use as an assessment tool is an improvement on standard evaluation techniques.

This study was not without limitations. First, it was a single-center study with a relatively small sample size. Second, the study is cross sectional and was not designed to determine whether serial urinary lipocalin-2/creatinine measurements can predict the onset and progression of SLE nephritis.

In this regard, future investigations on serial measurement of urinary lipocalin-2 together with its correlation with disease activity and treatment response will be needed. 

## Conclusion

 An important clinical conclusion is that adding measurement of urinary lipocalin-2 to the routine follow-up of LN patients, particularly those with biopsy-proven disease, may result in earlier diagnosis of lupus nephritis, and therefore less delay in institution of appropriate treatment. Thus, our results are important as they suggest a novel approach to the clinical management of lupus patients. Possibly, determination of urinary lipocalin-2 can be helpful in the evaluation of lupus nephritis in general.

 In conclusion, in the current study we found that urinary lipocalin-2 may act as a useful marker of lupus nephritis and provide additional clinically relevant information about disease activity to that given by the established marker.

## References

[1] Mok CC (2010). Biomarkers for lupus nephritis: a critical appraisal. J Biomed Biotechnol.

[2] Fiehn C (2006). Early diagnosis and treatment in lupus nephritis: how we can influence the risk for terminal renal failure. J Rheumatol.

[3] Cross J, Jayne D (2005). Diagnosis and treatment of kidney disease. Best Pract Res Clin Rheumatol.

[4] Liu CC, Manzi S, Ahearn JM (2005). Biomarkers for systemic lupus erythematosus: a review and perspective. Curr Opin Rheumatol.

[5] Bajaj S, Albert L, Gladman DD, Urowitz MB, Hallett DC, Ritchie S (2000). Serial renal biopsy in systemic lupus erythematosus. J Rheumatol.

[6] Mishra J, Ma Q, Prada A (2003). Identification of neutrophil gelatinase-associated lipocalin as a novel early urinary biomarker for ischemic renal injury. J Am Soc Nephrol.

[7] Devarajan P (2008). Neutrophil gelatinase-associated lipocalin (NGAL): a new marker of kidney disease. Scand J Clin Lab Invest Suppl.

[8] Siew ED, Ware LB, Gebretsadik T (2009). Urine neutrophil gelatinase-associated lipocalin moderatelypredicts acute kidney injury in critically ill adults. J Am Soc Nephrol.

[9] Mishra J, Dent C, Tarabishi R, Mitsnefes MM, Ma Q, Kelly C (2005). Neutrophil gelatinase-associated lipocalin (NGAL) as a biomarker for acute renal injury after cardiac surgery. Lancet.

[10] Pitashny M, Schwartz N, Qing X, aili B, Aranow C, Mackay M (2007). Urinary lipocalin-2 is associated with renal disease activity in human lupus nephritis. Arthritis Rheum.

[11] Brunner HI, Mueller M, Rutherford C (2006). Urinary neutrophil gelatinase-associated lipocalin as a biomarker of nephritis in childhood-onset systemic lupus erythematosus. Arthritis Rheum.

[12] Bolignano D, Coppolino G, Campo S (2008). Urinary neutrophil gelatinase-associated lipocalin (NGAL) is associated with severity of renal disease in proteinuric patients. Nephrol Dial Transplant.

[13] Mori K, Lee HT, Rapoport D, Drexler IR, Foster K (2005). Endocytic delivery of lipocalinsiderophore-iron complex rescues the kidney from ischemia-reperfusion injury. J Clin Invest.

[14] Brunner HI, Mueller M, Rutherford C, Passo MH, Witte D, Grom A (2006). Urinary neutrophil gelatinase-associated lipocalin as a biomarker of nephritis in childhood-onset systemic lupus erythematosus. Arthritis Rheum.

[15] Rubinstein T, Pitashny M, Putterman C (2008). The novel role of neutrophil gelatinase-B associated lipocalin (NGAL)/ lipocalin-2 as a biomarker for lupus nephritis. Autoimmun Rev.

[16] Koura HM, Galal A, Elshamaa MF, Kandil DM, Eman A, Elghorori, Eman S, Khalifa (2011). Urinary neutrophil gelatinase – associated lipocalin as a marker of disease activity inpatients with lupus nephritis. Int J Acad Res.

[17] Suzuki M, Wiers KM, Klein-Gitelman MS (2008). Neutrophil gelatinase-associated lipocalin as a biomarker of disease activity in pediatric lupus nephritis. Pediatr Nephrol.

